# Supplementary material for: Sleep Habits, Academic Performance and Health Behaviors of Adolescents in Southern Greece
Source: Healthcare (Basel). 2024 Apr 3;12(7):775. doi: 10.3390/healthcare12070775 (PMC11011514; doi:10.3390/healthcare12070775)
Supplement: Supplementary file 1 [file healthcare-12-00775-s001.zip › healthcare-2887119-supplementary.pdf]

Supplementary Material

Table S1. Sleeping hour of students during working days at 6 different classes, Post-hoc analysis p < 0.001:significant different .

(I) Classs	*p* (J) Classs					
	**Gymnasium 1st**	**Gymnasium 2st**	**Gymnasium 3rd**	**Lyceum 1st**	**Lyceum 2nd**	**Lyceum 3rd**
Gymnasium 1st		0.531	**0.008**	**<0.001**	**<0.001**	**<0.001**
Gymnasium 2st	0.531		1.000	**0.003**	**<0.001**	**<0.001**
Gymnasium 3rd	0.008	1.000		1.000	1.000	**0.018**
Lyceum 1st	<0.001	0.003	1.000		1.000	**0.003**
Lyceum 2nd	<0.001	<0.001	1.000	1.000		0.067
Lyceum 3rd	<0.001	<0.001	0.018	0.003	0.067	

Table S2. Sleeping hour of students during weekend days at 6 different classes Post-hoc analysis $p < 0.001$: significant different.

(I) Classs	Sig. (J) Classs					
	**Gymnasium 1st**	**Gymnasium 2st**	**Gymnasium 3rd**	**Lyceum 1st**	**Lyceum 2nd**	**Lyceum 3rd**
Gymnasium 1st		**1.000**	**<0.001**	**<0.001**	**<0.001**	**<0.001**
Gymnasium 2st	1.000		**0.016**	**<0.001**	**<0.001**	**<0.001**
Gymnasium 3rd	<0.001	0.016		1.000	1.000	1.000
Lyceum 1st	<0.001	<0.001	1.000		1.000	1.000
Lyceum 2nd	<0.001	<0.001	1.000	1.000		0.931
Lyceum 3rd	<0.001	<0.001	1.000	1.000	0.931	

Table S3. Health habits by age

		**13**	**14**	**15**	**16**	**17**	**18**	**19**	*p*
		***n* (%)**	***n* (%)**	***n* (%)**	***n* (%)**	***n* (%)**	***n* (%)**	***n* (%)**	
Smoking	Never/Rarely	114 (97.4)	88 (100)	45 (95.7)	206 (90.4)	171 (88.6)	123 (81.5)	3 (42.9)	<0.001
	Sometimes	2 (1.7)	0 (0)	1 (2.1)	18 (7.9)	13 (6.7)	21 (13.9)	1 (14.3)	
	Often	1 (0.9)	0 (0)	1 (2.1)	4 (1.8)	9 (4.7)	7 (4.6)	3 (42.9)	
Drinking Alcohol	Never/Rarely	105 (89.7)	79 (89.8)	39 (83)	110 (48.2)	66 (34.2)	42 (27.8)	1 (14.3)	<0.001
	Sometimes	11 (9.4)	8 (9.1)	8 (17)	115 (50.4)	121 (62.7)	106 (70.2)	4 (57.1)	
	Often	1 (0.9)	1 (1.1)	0 (0)	3 (1.3)	6 (3.1)	3 (2)	2 (28.6)	
Exercise	Never/Rarely	10 (8.5)	11 (12.5)	2 (4.3)	37 (16.2)	40 (20.7)	50 (33.1)	4 (57.1)	<0.001
	Sometimes	17 (14.5)	17 (19.3)	14 (29.8)	59 (25.9)	57 (29.5)	61 (40.4)	1 (14.3)	
	Often	90 (76.9)	60 (68.2)	31 (66)	132 (57.9)	96 (49.7)	40 (26.5)	2 (28.6)	
Coffee and caffeine product	Never/Rarely	91 (77.8)	73 (83)	32 (68.1)	122 (53.5)	120 (62.2)	51 (33.8)	2 (28.6)	<0.001
	Sometimes	20 (17.1)	13 (14.8)	11 (23.4)	80 (35.1)	44 (22.8)	54 (35.8)	3 (42.9)	
	Often	6 (5.1)	2 (2.3)	4 (8.5)	26 (11.4)	29 (15)	46 (30.5)	2 (28.6)	

Table S4. Adjusted ORs with 95%Cis of sleeping ≥8 hours per day on students' grades.

Grades (18+)	OR	95% C.I.for OR		p
		LL	UL	
**Females**	**1.84**	1.39	2.43	**<0.001**
Lyceum	0.73	0.54	0.99	**0.045**
Sleeping ≥8 hours	1.48	1.06	2.07	**0.022**
